# Culinary Solitude in the Diet of People with Functional Diversity

**DOI:** 10.3390/ijerph19063624

**Published:** 2022-03-18

**Authors:** Carmen Cipriano-Crespo, Francesc-Xavier Medina, Lorenzo Mariano-Juárez

**Affiliations:** 1 Faculty of Health Sciences, University of Castilla La Mancha, 45600 Talavera de la Reina, Spain; mariacarmen.cipriano@uclm.es; 2 Faculty of Health Sciences/Foodlab & Unesco Chair on Food, Culture, and Development, Universitat Oberta de Catalunya (UOC), 08018 Barcelona, Spain; 3 Faculty of Nursing and Occupational Therapy, University of Extremadura, 10003 Caceres, Spain; lorenmariano@unex.es

**Keywords:** functional diversity, commensality, self-esteem, shame, loneliness

## Abstract

This qualitative ethnographic study identifies how problems in the feeding process of a group of people with functional diversity influence different eating situations. The study, which was carried out in the Autonomous Community of Castilla La Mancha, Spain, is based on interviews conducted at the headquarters of the different participating associations for functionally diverse people, at the participants’ homes, and in public spaces. The study included 27 subjects aged between 18–75 years. Their functional diversity had caused significant changes in their sociability, particularly in contexts associated with food consumption. The analysis identified three main themes: social ghettoisation and culinary loneliness; stigma, shame, feeling like a burden, and loneliness; and exclusion or self-exclusion at the dining table. Our participants’ narratives underscored the importance of acknowledging the significance of changes in eating-related sociability due to functional diversity. For the study subjects, grief, loneliness, and shame contributed to disassociating food consumption from social celebrations, withdrawing from restaurant meals, or conversations while eating to avoid other people’s stares.

## 1. Introduction

Eating is an essential aspect of life—a basic component of the daily activities of social groups, and a cornerstone of the biological, psychological, and cultural characterization of human beings [1]. From the viewpoint of anthropology, eating is a key dimension of social relationships—encompassing much more than the consumption a certain number of nutrients based on biological or dietary considerations [2]. As Contreras [3] notes, eating is a social act and a key aspect for the creation of cultural identities. Eating together involves participating in a social activity where attitudes, protocols, behaviors, and situations are shared with others [3]. It is an emotional act that allows us to draw symbolic nourishment from the values represented by different foods [4], and it is closely intertwined with personal well-being and the happiness derived from sitting at a table with others. As Spanish chef Carme Ruscalleda notes: “*Cooking is like embracing someone: it creates happiness*” [5].

As eating is crucial for our physical and social development, eating in the company of others is an essential aspect of creating and maintaining a sense of community. When food is consumed with others, the personal, intimate act of eating is transformed into a shared experience—a collective experience. Thus, the dining table can be perceived as a scenario in which the relationships—kinship, friendship—between those sitting together are reproduced, and common traditions, tastes, and pleasures are displayed [6,7].

As a result, the practice of eating together is a basic component of our social nature. [8]. As Medina [7] has suggested, commensality is more than sharing food and spending time together—its final goal is the preservation of the social structure of a group. Flandrin and Montanari [9] suggest that the difference between human beings’ culinary behavior and animal eating practices lies in culture, the methods of food preparation, and the social role of food consumption.

As Levi-Strauss [10] notes, food is closely intertwined with social relationships—not only does it provide nourishment, it also allows us to transmit emotions and feelings. While we share food with others, we communicate, and we reminisce about the past. While we eat, we converse, get closer to others, and establish bonds. Indeed, as Maffesoli [11] suggests, “most of the time, human beings prefer to eat in the company of others—conveying feelings and thoughts and communicating problems and different kinds of situations”.

Eating together creates a spatial and temporal convergence among individuals who share a common bond. Commensality requires interaction among the members of a group—to gather in a specific place at a particular time to eat together, thus engaging in a shared practice. This reinforces the symbolic feeling of belonging to the group that the table is shared with [12]. At the same time, the practice of eating together can have different meanings for the people involved, depending on their gender, age, their role within the social group, and their social and cultural position. For some, it might be just one of their day-to-day obligations—they might sit at the table while watching television, oblivious to the social dimensions of commensality [13].

Functional diversity is an alternative term for “disability” that aims to avoid the negative connotations of this concept. To acknowledge each individual’s diversity fosters a richer society—one that recognizes that this is an inherent dimension of the human condition and that, indeed, each individual can be considered functionally diverse [14]. However, functional diversity can substantially impact different spheres of life, including nutrition. Grignon described a “segregative commensality”, with eating and drinking as a way of reinforcing and/or rejecting the social group. Accordingly, those who are perceived as alien or different because of their eating practices might experience social distancing. Our study explored the major difficulties experienced by our participants in the eating process—difficulties that affected these situations of social commensality and caused social distancing and eating in situations of loneliness.

Rather than enduring those uncomfortable situations, our participants opted for social (self) isolation—a progressive distancing from social situations involving food consumption such as restaurants and other social events—as they felt that functional diversity prevented them from performing adequately. As a result, they preferred to eat alone, out of sight from others. Although eating alone is often associated with a reduced enjoyment of life, for those experiencing eating difficulties, it was a better alternative [15]. At the same time, these situations of isolation they lived with, the feeling of being deprived when compared to others, increased their feelings of loneliness—as often happens to people with functional diversity, who feel their personal situation is somehow deficient [16]. This has been noticed often in people with dysphagia, who experience social limitations during mealtimes due to difficulties swallowing and maintaining conversations while eating. In these situations, eating with their families can be an element of friction rather than the source of social pleasure that commensality is supposed to be [15].

From childhood to adulthood, human beings are influenced in their eating habits by their surrounding society. Other people’s behavior provides guidelines for our own—making us act, at the very least, in the most appropriate way according to the social rules within a given group [17]. For instance, although food consumption always has a social dimension—the dining table being, by definition, a space for social interaction—this is more important than ever in exceptional situations of commensality that include extended family or friends. Eating is an act that allows us to integrate into society: “we eat according to certain standards that translate into permissions and prohibitions regarding times, places, and table manners; and we invite others to our table to share, negotiate, flaunt, or dominate” [18].

In this sense, eating incorporates a series of material or immaterial dimensions closely associated with the participants themselves, including following a certain number of explicit or implicit rules [8]. However, many people experiencing eating problems due to functional diversity are unable to comply with these rules and find themselves rejected by individuals who do not experience these problems, eventually deciding to eat alone.

This study proposes an ethnographic approach to the eating experience of a group of people with different types of functional diversity that caused eating difficulties. These problems sometimes made them feel that they did not belong within the group on which they had built their social identity. Although eating before functional diversity was a source of enjoyment, the difficulties it entailed in their present situation contributed to their perceiving food as a stress factor—an activity that required extra effort and where social and sensorial satisfaction was denied. The experience becomes incomplete because neither food nor the experience of food consumption is shared with others. On the contrary, the relationship between food and functional diversity only led to the emergence of feelings of shame and loneliness.

## 2. Materials and Methods

This is a qualitative ethnographic study of subjective experiences of commensality among a group of functionally diverse people. Data collection and analysis followed an inductive approach [19,20,21]. Several categories were established from the analysis of the empirical materials, based on constant comparison and grounded theory methods [22,23]. This provided a better understanding of our participants’ experiences of challenges associated with food consumption [23,24].

On the other hand, this study was conceived from the viewpoint of the anthropology of food, whose methods have their own characteristics, perspectives, and applications [25,26,27,28].

### 2.1. Participants

This study included a total of 27 participants, aged between 18 and 75 years. All were functionally diverse people, with different functional deficiencies—total or partial—that caused challenges related to eating and drinking—i.e., cancer, spinal cord injuries, Niemann–Pick disease, duchenne muscular dystrophy, multiple sclerosis, amyotrophic lateral sclerosis, ictus, or acquired brain damage (Table 1). The main factor behind the selection of each participant was, above all, the researchers’ accessibility to the field.

Additionally, all participants experienced eating difficulties due to the presence of functional diversity. Some of our participants presented with difficulties chewing or swallowing food, whereas others struggled to use the implements necessary to feed themselves. Consent for the interviews being audio-recorded and signing the informed consent form were essential prerequisites for participation in the study. Individuals with dementia and/or severe cognitive impairment, those who did not provide informed consent, or those who refused to have their interviews audio-recorded were excluded from the study.

Potential participants were approached through different channels. The project was introduced to the heads of different associations for functionally diverse people, who recommended suitable participants who were experiencing eating difficulties. We also contacted occupational therapy professionals at the National Hospital for Paraplegics (Toledo, Spain) who, after obtaining approval from the head of the department, facilitated contact details of patients with eating problems.

### 2.2. Data Collection

Data were collected through semi-structured, in-depth interviews that followed a pre-established guide (Table 2). This allowed new themes to emerge and be explored during the course of the interviews [29,30]. All the interviews were audio-recorded.

Most of the interviews were conducted in designated areas at the headquarters of different associations for functionally diverse people, although some took place at the participants’ homes or in public spaces. Empirical data were also collected through participant observation in settings such as kitchens or canteens. Ensuring the participants’ comfort and privacy during the interviews was always a priority. To guarantee privacy, each audio recording was assigned a coded name, and any personal data revealing the participants’ identity has been removed from this article. The names used in the text are fictitious; they have been added to make the text easier to read and humanize the context. The original recordings were destroyed once the interviews were transcribed. All interviews were conducted by the three members of the team, all of whom had extensive experience in qualitative research. Interviews had durations of between 60–120 min.

### 2.3. Data Analysis

The interviews were transcribed verbatim by the researcher who conducted them. Data analysis used Microsoft Access and was performed by two experienced qualitative researchers, who worked independently. The results were shared and discussed once their analyses were completed. The participants’ responses were used to create a thematic map with different themes and subthemes.

Each participant received an alphanumeric code that was used for logging data and creating categories, and as a reference for literal quotations extracted from the participants’ interviews.

The study followed the items defined in the COREQ checklist for reporting qualitative research [31] and Lincoln and Guba’s quality criteria framework [32].

### 2.4. Ethical Considerations

The study was approved by the Clinical Research Ethical Committee of the Talavera de la Reina Integrated Management Area (CEIm del AGI de Talavera de la Reina, Spain, Hospital Nuestra Señora del Prado, ref: 18/2014). The study was conducted in accordance with ethical principles outlined in the Declaration of Helsinki and the Belmont Report. Data were treated in line with current guidelines on the ethical implications of research. Data were used with the utmost confidentiality, and remain protected under the Law 15/1999, of December 13, on Protection of Personal Data.

## 3. Results

Functional diversity causes significant challenges and transformations in the life situations of the people affected at very different levels—physical, social, and emotional. It also creates challenges related to eating—with changes in the way food is consumed affecting commensality and sociability.

The analysis of empirical materials revealed three recurrent themes that highlight the importance of commensality for people with functional diversity.

### 3.1. Social Ghettoisation and Culinary Loneliness

Some of the participants in our study mentioned withdrawing or being socially excluded by people without eating problems, thus triggering feelings of “culinary loneliness”. In a way, this exclusion provided a certain sense of comfort that was impossible when eating while being subjected to other people’s scrutiny. In their accounts, our participants mentioned avoiding eating out in restaurants or deciding to eat on their own—without being stared at, but also without other people’s company. Eventually, this fostered a process of culinary ghettoization and a withdrawal from public spaces—the visible space, without culinary limitations, that used to be enjoyed in restaurants. One participant, Paloma, who lived in a care home for older adults due to limitations caused by amyotrophic lateral sclerosis, noted that she often asked assistants to place her with her back to other residents during special celebrations such as birthdays or Christmas.

*“[…] leave me in my little corner so I can eat a bit of cake on my own, with nobody looking at me in disgust.”*

Social exclusion and isolation due to problems in the eating process are not an issue when food and drink are consumed within a social environment in which our participants feel included—i.e., when eating with people with similar difficulties and who belong to the same associations as them. This helps normalize their experience and increases the opportunities to re-establish lost relationships based on sociability and commensality. In these environments, their experiences are normalized, and they do not feel questioned in their social interactions. One subject, Fernando, described his experience:

*“When I go out with friends I feel embarrassed that I will spill food down the sides, because people stare at me; but when I attend the meals organised by the association, which I love doing—I do not mind if one of my buddies asks me to wipe something that I’ve dropped, he has been through the same as me, and I do not need to justify anything.”*

Isaac, another participant, decided to go out less often due to his increasing difficulties eating and speaking, caused by multiple sclerosis. Before that, he enjoyed meeting friends for lunch or dinner, since it was a perfect excuse to catch up with them. Now, however, he did not attend these meetings. Instead, he ate with his parents—but only because he had no other option, always keeping the television on to avoid interacting with them.

*“I have nothing to talk about; before, there were plenty of subjects, but now everything revolves around my illness, and I rather not speak.”*

Our participants tended to look for people experiencing similar challenges to theirs, so they could feel understood in what they were going through. They needed to share their feelings, emotions, and experiences, instead of feeling rejected due to their problems. A small number of participants admitted sometimes eating alone, in quiet corners in restaurants, looking at a television, and withdrawing from social conventions and contact with others—thus breaking implicit rules of social communication. On the other hand, when people eat alone, what they eat is of less consequence—they can eat whatever is there. Additionally, the amount of time devoted to eating also decreases. The practice of eating loses its social dimension and is reduced to satiating a physical need, which only takes a few minutes. However, eating alone and in such a short amount of time is another way of breaking with normality.

### 3.2. Burden, Shame, and Loneliness

To eat in society necessarily implies a certain degree of interaction with other people—mutually passing each other food, serving beverages, sharing an experience. Each of those attending a family or work meal are crucial threads of the social mechanism that contributes to creating pleasure and enjoyment for themselves and other people.

Our participants, by contrast, described feeling like a burden, a nuisance that got in the way of the logical order of things. Gradually, certain thoughts, feelings, and ways of acting emerged, reflecting the social isolation and monotony that, they felt, had been imposed upon them.

This feeling of being a burden was clearly associated with the shame experienced during social interaction in food-related contexts. For instance, those participants who had undergone a laryngectomy reported drastically reducing their participation in collective gatherings that involved food being served and consumed, due to their feeling shame and being a hindrance to everybody else.

Paloma understood very well this feeling of being ashamed. In the care home where she lives, she eats in a room on her own, at a different time from other residents, with only the company of the assistant who helps her.

*“Initially, I used to eat with everybody else, but while I eat, I drop a lot of food, and once I heard a man saying aloud that it was disgusting, and he turned his back on me so he would not see me. Since that day, I prefer to eat when I am alone.”*

Paloma described feeling ashamed of eating in front of other people, facing their looks and comments. Consequently, she made a decision that also implied giving up on certain things—for instance, due to her difficulties swallowing, her food always cooled down, and she had to eat it like that.

*“Since the kitchen is too far from the room where we eat, they do not warm [the food] to avoid wasting time, and when I have asked for a microwave to be brought close by they’ve said that this was not a dining room—so it is either eating cold food or going back to eating with the others, and I do not want to go through that again.”*

The feeling of shame could also be triggered by sharing a table with people who were not familiar with the difficulties they were currently experiencing. For example, María, who has muscular dystrophy, felt ashamed of not being able to eat as before with those who were part of her life back when she did not need any help to eat, and who still remembered her as she used to be. They were aware that she had a neurodegenerative disorder, but they had not seen its actual impact on her.

*“[…] I remember the day I met for dinner with a childhood friend, I felt like a stranger by her side despite her being aware of my illness because we had lived together—it was like going back in time and being compared with my younger self. With the people I see on a day-to-day basis, everything is normalised, there is feedback, so often I do not need to ask for anything—with a look or even my posture they know whether or not I want a drink, or a bite of some of the food at the table. They have assumed this gradual neurodegenerative process as naturally as I have. However, I find it a bit harder with those who were part of my life when I was better, it triggers these internal emotions.”*

Another participant, Isaac, with multiple sclerosis, also mentioned feeling ashamed when he spilt the food he was eating. His frustration over this made him eat less, particularly those foods that required using a spoon—which used to be his favorite. He used thick straws to eat soup without spilling it, but only at home—he was ashamed of being seen eating like a small child.

Progressively, this feeling of shame acquires new layers of meaning, eventually causing them to make difficult decisions—such as not serving or eating food in any celebration. This, however, causes further distancing and isolation from the people who do. Javier, who underwent a laryngectomy due to laryngeal cancer, told us that his eating difficulties had changed everything.

*“Because of my issues, we do not celebrate anything, only my children’s birthdays. We do not celebrate anymore because everybody knows that not being able to eat like them pains me, so to avoid it we do not celebrate with food, which is what we used to do in my family—like almost everybody else does, this is Spain! And here we like to eat and drink well.”*

The social stigma attached to people with functional diversity disrupts the consumption of food with other people in public spaces. The stares, the rejection, and sometimes even being denied service at certain restaurants make it almost impossible to share a table with others. As a result of these experiences, they feel lonely in their daily routines. This loneliness, however, might be perceived rather than real. This was the case with one of our participants, Isaac. His subjective perception was that he was lonely, despite being surrounded by people who cared about him. His parents and friends constantly knocked at his door to ask how he was feeling, to make him feel supported in this process he had to live with. However, he could not—or did not want to—feel comforted by this. Isaac admitted that his behavior towards others changed when he faced the unstoppable advance of his illness, and that this might have been the reason why his relationship with his wife soured.

*“My wife and I started bickering a lot because she did not understand what I was going through—she did not understand that I felt lonely despite her being with me, until one day she left me. It’s just that this that I have—it is only half a life, and badly lived at it.”*

The negative experiences cause grief and yearning for their former selves, feeding the stigma attached to the new culinary situation of those who bring functional diversity to the dining table.

### 3.3. Exclusion or Self-Exclusion from the Table

When sitting around a table laden with food, we express our internal self, concerns, and differences with those with whom we share the table. In this context, those obstacles that might challenge or disrupt commensality quickly become apparent. In our study, these were associated with our participants’ new dietary needs and how they consumed food or drink now—challenges caused by an illness or injury, which turned people with functional diversity into *outsiders* at the table.

For this reason, sometimes, they choose to withdraw from these situations—for fear that their presence will subvert the unspoken hygiene rules that guide table manners, that dictate that food has to be attractive and people have to enjoy each other’s company.

For instance, regarding social conventions and habits established around communal dining, several participants with cancer noted that they could not follow the normal rhythm of food consumption or conversation—since they needed to eat slowly and concentrate on chewing to avoid choking. Fear of receiving puzzled stares, of the noises they might make, or of expelling tracheal mucus while eating were some of the reasons expressed to justify their feeling excluded or their decision to withdraw from such events. One participant, Leoncio, expressed it thus.

*“I do not want to eat out with friends or relatives, food dribbles down my sides, and I notice people staring at the hole in my throat—I feel ashamed of what I have—and when I do not eat out, I do not suffer.”*

Another participant, Florencio, mentioned eating out with friends occasionally but always in quiet places because otherwise he had to remain silent—since it upset him if nobody could understand what he was saying. Indeed, he did not often feel like eating out with large groups of people. He self-excluded because he felt different—always making demands that were not necessary for others, but they felt obliged to comply with.

*“Besides, sometimes in the heat of the moment everybody raises their voices too much and drown my own, so I have to remain silent and just listen for the rest of the meal.”*

Both of these participants pointed out that the negative feelings they experienced made them withdraw from sitting at a table and sharing food with people who did not have any problems. In many cases, in the absence of individual or collective mechanisms specifically focused on their needs, people with functional diversity end up eating alone—a clear sign of social exclusion. As Jesús, with laryngeal cancer, pointed out: “*This is an illness that excludes you from society*”.

For people with functional diversity, self-imposed exclusion at mealtimes is primarily due to the stares they receive from others. Israel, with Duchenne muscular dystrophy, gradually severed his relationships with others as his illness progressed. His eating habits were gradually modified to adapt to the loss of manual dexterity in both hands. However, these new habits were not socially accepted by people *on the other side* of the functional diversity divide. Thus, he preferred to consume food in private—in the intimate environment of his dining room.

*“People are surprised when they see me eating—I get very close to the plate and sometimes food dribbles down the sides; eventually my mother has to feed me and, at my age, I do not like people seeing me as a small child.”*

María also decided to exclude herself from situations in which she had to eat with other people because of her eating difficulties. Recently, attending the wedding of a close relative, she asked to be served food on her own, before other guests arrived at the restaurant—protected from prying eyes but consequently excluded from social engagement. This self-imposed exclusion meant giving up on exchanging opinions on the food served—flavors, textures, and recipes in which, as a cook, she was particularly interested. Instead, she chose to contemplate others while they enjoyed the flavors, textures, and presentation of the different dishes, finding comfort in not being scrutinized because of the way she ate.

Self-exclusion might also be caused by experiencing dysphagia problems, as was the case of Benito as a consequence of laryngeal cancer. He minimized his attendance at family birthday celebrations, despite his wife always carrying in her bag some fluid thickener to add to his drink. However, the constant coughing caused by drinking made him stop attending celebrations that involved food consumption.

*“It looks like I am going to die, I get very red and cough a lot, scaring everyone around me. Besides, sometimes I dribble down the sides when I am drinking, and I get covered in stains, and people notice and give me weird looks.”*

Again, other people’s stares made our participants want to *hide* when they were eating.

### 3.4. Condemned to Distance, Loneliness, and Ugliness

Some of the study participants had to be tube-fed, and their perception of their situation was even more dramatic. As Jesús described, “*I thought that I was going to die—that I was at the end*”.

For the people needing feeding tubes, the self-perception of their physical integrity is impaired. An exploration of tube-fed patients noted that some of them felt ugly [33]—despite most patients admitting that they were necessary, tubes were perceived as a negative element that disrupted the “normal” aesthetic image of an individual.

Most of our tube-fed participants reported experiencing a certain degree of rejection. As Isaac noted, “When something like this happens that is forever, people turn their backs on you—they do not want to know much about the course of the illness, they run away from grief”.

These feelings of loneliness are closely related to sadness at being distanced from a world that once included them, leaving a gap that is sometimes filled with people with similar experiences to theirs—the only ones who could understand what they were going through. For instance, Samuel had a rare disease. His mother, Isabel, faced a lack of understanding from people unaware of the amount of suffering that an eventual diagnosis and the progression of the disease could cause. With a voice tinged with sadness, she told us:

*“We gave our everything to the association—in there, there were no excuses and no lies, we all felt abandoned by the healthy ones and understood that they could not cope with the disease—people don’t want grief.”*

There was no need to explain anything in the association she became a member of. Sooner or later, everybody went through the same experiences, which made them all equal despite their individualities. She felt it was important to give visibility and create social awareness of her son’s disease, and she did this with the association’s support—without it, she would not have been strong enough.

Initially, friends and relatives visit the person with functional diversity regularly. However, as time goes on, visits become less and less frequent. Isabel remembers that her friends stopped calling because they could not endure the image of her son, sitting in his chair, absently watching cartoons. “*At first, they used to phone me and say, ‘I could not cope with what you are going through!’ But eventually, the phone calls became few and far between, until one day the phone stopped ringing.*”

Those participants who moved back permanently to their homes after a period at a hospital soon had to face a new reality: other people had busy lives. As a result, their opportunities for social engagement were often sharply reduced—since they depended on others to go out for walks or eat out in restaurants. Arturo, who has a spinal cord injury, told us that “*My friends are keeping an eye on me, they are shocked by what happened to me—they have to come and pick me up, take me places, and sometimes they are not available when I would like them to, but until I get a license and I can drive myself I have no choice but to rely on them*”.

This constant reliance on others to participate in gatherings or special meals dramatically increases the plight of people with functional diversity and their relatives. It makes them even more aware of everything that has changed and what they have lost due to their new life situation.

The realization that these new circumstances are permanent, as was the case of the study participants, might make them feel condemned to loneliness—a feeling that they attributed to their weakness and the grief caused by their inability to cope with their difficulties, which affected their self-esteem. Indeed, some of the participants clearly stated that their withdrawal from food-related events was due to their eating difficulties—so they did not make food *ugly*. They preferred not to display their challenges to those they shared the table with and assume the price to be paid for this—an increasingly reduced sociability. This not only affects them but also, indirectly, their closest relatives—who sometimes are unable to participate in social events either as a result. That was the case with Oliva, Paco’s wife. Paco had acquired brain damage, which caused difficulties in eating without spilling food and swallowing without choking. Oliva felt she had been forced into culinary loneliness due to his decision to reduce his participation in situations of commensality. With tears in her eyes, she described that because of Paco’s eating and communication problems, her only social interaction was with their son and with the healthcare assistant who helped them at home. “*I am having to pay the price—because he does not want to go out anywhere, I am doomed to stay at home all day—it’s like being locked for life, just because he is ashamed of eating in front of others.*”

However, there are also situations in which feelings of anxiety and fear are counterbalanced by the willingness to experience the pleasure of social engagement. That was the case of Javier who, despite requiring artificial feeding, fought his fears to be able to enjoy participating in situations of sociability. “*I play a round of cards with my friends and, very slowly, I drink a shot of gin—real slow, it can last me the whole afternoon. All of my friends were drinking and I could not stand it anymore. I felt like a third wheel.*”

The loss of independence and intimacy gives rise to a whole host of new emotions—due to the shame of not being the same as they used to be before, and other people noticing the difficulties they experienced in conducting simple, day-to-day tasks. This process made them feel lonely, despite the support and involvement of relatives and professionals in important moments of their lives.

## 4. Discussion

Our analysis revealed the importance of loss of food-related sociability for people with functional diversity due to the eating challenges they experience. These challenges impacted our participants’ self-esteem, as they equated the loss of their physical abilities with their bodies losing their value. Their bodies were different from before, no longer fit for purpose. They were no longer legitimate, productive, independent bodies. Instead, they were *disabled bodies* [34]—which, for our participants, meant the loss of their symbolic capital. The increasing limitations in their manual dexterity were translated into the loss of their ability to engage with other people through food consumption.

As noted in previous studies [35,36], the loss of social interaction in this kind of context is a frequently mentioned issue. People with functional diversity are aware that the experience of culinary loneliness will be there for the rest of their lives, which is detrimental to their mental health [37].

For our participants, functional diversity meant losing opportunities to derive pleasure from situations of commensality that were a source of enjoyment before—as experiences shared with their loved ones. This loss affects not only the people with functional diversity, but also those who share their lives—in many cases, through self-imposed exclusion, due to their perceived inadequacy when eating in front of others in work or family social gatherings. Studies such as those of Winkler have pointed out that human interactions help define personal identities [38,39]. For people with functional diversity experiencing eating and drinking challenges, spaces and opportunities for sociability are reduced, thus affecting their personal identity.

Most people feel the importance of belonging to a wider social sphere—a group, a family—and to be recognized as individual members of their community. The practice of eating together as a group has a normative dimension—there are some common rules and expectations from those participating in a situation of commensality, and those who do not abide by them are excluded [40]. However, as described above, our participants experienced difficulties maintaining these standards due to their new life situation. This disrupted the balance required to follow the internal rules guiding the social groups to which they belong. To help maintain a harmonious environment where they could feel comfortable, people with functional diversity tend to withdraw and exclude themselves. Consequently, they deny themselves the right to participate and derive enjoyment from previously pleasurable situations of commensality. When they eat, it is out of necessity—to receive nourishment. Their functional diversity does not allow them to experience enjoyment in relation to food. Instead, food is perceived as a requirement, something that has to be consumed to reach specific dietary goals without providing any pleasure [41]. As a result of subjective perceptions of external attitudes, our participants consider themselves useless—a burden, an affront to others—and choose instead to withdraw [42].

The results of this study have relevance for clinical practice. In general, intervention models related to food and drink intake for people with functional diversity who experience eating difficulties tend to focus on the nutritional aspects of the process. However, the stories analyzed in this study underline the importance attributed to the social and symbolic dimensions of the eating process. At the same time, the study provides a detailed examination of the consequences of the new food situation on contextual relationships and culinary loneliness, all of which can be detrimental to mental health [37].

This study has suggested a possible approach to explore the experiences of people with functional diversity. However, an important limitation is that the participants presented very different kinds and levels of functional diversity, caused by a wide range of pathologies.

Another potential limitation is that the study did not assess how age and gender affected these experiences. Our main aim was to explore the impact of different types of functional diversity on eating practices and how this was perceived. However, a gender-based approach could help explore whether these situations were differentially experienced and assess possible male/female variations in meaning.

Finally, it would also be interesting to explore how cooking and eating aid equipment—e.g., cutlery with special handles, etc.—can mediate these subjective experiences.

## 5. Conclusions

Ethnographic evidence gathered in our study revealed how functionally diverse people progressively reduced their participation in situations of social commensality—either attending fewer events or reducing the number of people present at a time—thus modifying conditions to access and derive pleasure from food consumption. Our study also revealed the importance of food for the construction of society—particularly the emotional and social relationships established around shared food consumption. The evidence emerging from the exploration of culinary sociability suggests that the loss of physiological function has a direct impact on the loss of the social value attached to food and food consumption.

Spinal cord injuries, the progress of neurodegenerative disorders, or the consequences of laryngeal cancer, among others, are factors that prevented our participants from enjoying eating and drinking together with other people—which eventually led them to perceive food only as a dietary requirement.

In contexts involving people other than their closest personal relationships, most of our participants expressed feeling ashamed of the difficulties they experienced or the way in which they now had to eat or drink. To avoid experiencing more grief regarding their personal situation, they placed limitations on the number of people they felt comfortable sharing situations of commensality with. This resulted in a gradual reduction in their sociability, and that of their closest relatives—whose opportunities to derive pleasure from social engagements were also altered and diminished.

## Figures and Tables

**Table 1:** Participants’ profiles and types of disability.

	Count (*n*)
Gender	
Female	6
Male	21
Disability	
Brain injury	6
Spinal cord injury	5
Cancer	10
Amyotrophic lateral sclerosis	1
Multiple sclerosis	2
Muscular dystrophy	2
Niemann–Pick disease	1

**Table 2:** In-depth interview guide: categories and questions.

Biography
Medical history, social and economic profile
Food and feeding
Feelings and emotions
Food and social activities
Acceptance of changes
Community and personal relationships
Successes, failures, exclusion
Friendship loss

## Data Availability

The data presented in this study, except those protected under confidentiality agreements with participants, are available on request from the corresponding author.
